# Laryngeal muscle tension in patients with sinonasal diseases: prevalence and clinical significance

**DOI:** 10.1017/S0022215124000847

**Published:** 2024-11

**Authors:** Abdul-Latif Hamdan, Jad Hosri, Yara Yammine, Patrick Abou Raji Feghali, Nadine El Hadi, Elie Alam

**Affiliations:** Department of Otolaryngology and Head & Neck Surgery, American University of Beirut Medical Center, Beirut, Lebanon

**Keywords:** laryngeal diseases, muscle tension pattern, rhinitis, sinusitis, laryngology

## Abstract

**Objective:**

To determine the prevalence of laryngeal muscle tension in patients with sinonasal diseases.

**Methods:**

The medical records and video-recordings of patients with a history of sinonasal disease were reviewed to identify one of four muscle tension patterns during phonation. A control group with no history of sinonasal diseases was matched according to age and gender.

**Results:**

Seventy-seven patients were divided into a study group (*n* = 47) and a control group (*n* = 30). In the study group, 29 patients had at least one muscle tension pattern compared with only 9 in the control group (*p* = 0.007). The most common muscle tension patterns observed in the study and control groups were muscle tension patterns II and III. In the study group, 79.3 per cent of patients with at least one muscle tension pattern reported dysphonia compared with only 33.3 per cent in the control group.

**Conclusion:**

Patients with sinonasal diseases are more likely to exhibit laryngeal muscle tension and dysphonia in comparison with healthy subjects.

## Introduction

The sinonasal tract is subject to a variety of diseases. These may be infectious, inflammatory, neoplastic or autoimmune. The clinical presentation of affected patients varies with the aetiology of the disease and its duration. The most commonly reported symptoms are nasal obstruction, rhinorrhoea, sneezing, lacrimation and post-nasal drip. More severe and impairing symptoms, such as facial pain and/or pressure, bleeding and hyposmia and/or anosmia, may also prevail.^1^^,^^2^

Sinonasal diseases are associated with many co-morbidities that have a detrimental impact on the physical, psychological and social well-being of patients.^3–5^ In a study that included 131 subjects with chronic rhinosinusitis, Hoehle *et al*. highlighted the adverse effect of sinonasal diseases on several health-related subdomains, including emotional dysfunction. The authors also noted a significant correlation between the Sinonasal Outcome Test 22 (SNOT-22) score and the EuroQol five-dimensional survey and visual analogue scale (*r* = −0.53, *p* < 0.001).^6^ In another cross-sectional analysis using National Interview Survey data, Zhou *et al*. noted a correlation between sinusitis and/or allergic rhinitis and sleep deprivation, depressive symptoms and number of workdays lost.^5^

Given the importance of voice as a means of communication and a source of income to one-third of the workforce,^7^ the impact of sinonasal diseases on voice became a subject of interest to many investigators. The focus has been on nasality because of the paramount role of the sinonasal cavities in sound amplification and resonance.^8^ The consensus is that patients with sinonasal diseases have hyponasality in comparison with patients with no sinonasal diseases. Hong *et al*. assessed the nasality of patients with nasal polyposis and noted significantly lower nasalance scores^9^ in comparison with healthy subjects.^10^ In another study that included 81 patients with chronic rhinosinusitis undergoing endoscopic sinus surgery, Jiang and Huang showed a moderate correlation between midnasal and postnasal volumes and nasalance score. This latter increased with an increase in nasal volumes.^11^ The impact of sinonasal diseases on voice has also been proven in patients undergoing endoscopic sinus surgery using different voice outcome measures, such as the Voice Handicap Index, Grade Roughness, Breathiness, Asthenia, Strain (GRBAS), fundamental frequency, jitter, shimmer, noise to harmonic ratio and formants scores.^12–22^

The laryngeal behaviour during phonation in patients with sinonasal diseases has not been previously described.^23–26^ The purpose of this investigation was to report the prevalence of laryngeal muscle tension in a cohort of patients with sinonasal diseases in comparison with a control group with no history of sinonasal diseases. The authors attempted to answer the following question: Are patients with sinonasal diseases more likely to exhibit laryngeal muscle tension compared with healthy subjects? The hypothesis set forth is that patients with sinonasal diseases are more likely to have laryngeal tension, and hence are more prone to develop voice disorders.

## Materials and methods

After having received the approval of the Institutional Review Board, the medical records and video-recordings of patients who presented to the rhinology clinic of a tertiary referral centre between 1 January 2020 and 30 June 2022 with history of sinonasal disease and who had filled the SNOT-22 questionnaire were reviewed. The SNOT-22 questionnaire is a self-reported outcome measure commonly used in the evaluation of various rhinological-related diseases.^27^ Only those with a SNOT-22 score above 7, indicating history of sinonasal disease, were included.^28^ Exclusion criteria included those with a SNOT-22 score below 7, patients with history of laryngeal surgery or manipulation and/or patients with history of upper respiratory tract infection. Professional voice users were also excluded in view of the confounding effect of high vocal loading. A control group with no history of sinonasal diseases was matched according to age and gender.

Demographic data included age, gender, history of smoking and symptoms such as nasal obstruction, nasal discharge, facial pain or headache and post-nasal drip. The video-recordings of the laryngeal examination of the study group and controls were reviewed by two otolaryngologists for the presence or absence of four muscle tension patterns, and inter-rater reliability was computed to assess the consistency of the reported findings. All subjects enrolled in this study had laryngeal examination as part of their work-up using the flexible nasopharyngoscope with distal chip camera (HD Video Rhino-Laryngoscope, Karl Storz, Tuttlingen, Germany). All patients were diagnosed with primary muscle tension without evidence of an underlying laryngeal pathology or neuromuscular disorder.

The muscle tension patterns were categorised according to the classification by Koufman *et al*:^29^ muscle tension pattern I is characterised by the presence of a gap between the vocal fold edges during phonation, muscle tension pattern II is characterised by medial compression of the false folds, muscle tension pattern III is characterised by an antero-posterior shortening of the distance between the petiole and the arytenoids, and muscle tension pattern IV is characterised by complete sphincter-like closure of the supraglottis during phonation. Secondary outcome measures included hoarseness, defined as a change in voice quality, globus sensation and repetitive throat clearing and/or cough. The presence of vocal fold structural abnormalities was also noted.

The authors assert that all procedures contributing to this work comply with the ethical standards of the authors’ institution's guidelines on human experimentation and with the Helsinki Declaration of 1975, as revised in 2008.

## Results and analysis

### Statistical analysis

Analyses were performed using Statistical Analysis Package for Social Sciences (version 25.0; SPSS, Chicago, IL, USA). Descriptive statistics were applied for different variable types. The chi-square test was used to determine the association between categorical variables. The independent *t*-test was used to determine the association between continuous variables. Data were represented as means ± standard deviation and a *p* value of less than 0.05 was considered significant. The video-recordings were reviewed by two otolaryngologists and inter-rater variability was computed to assess the reliability of the reported findings.

### Demographic data

A total of 77 patients were included in this study. The mean age of the total group was 43 ± 15 years, and the male to female ratio was 3.05. The patients were divided into two groups: a study group (*n* = 47 cases) and a control group (*n* = 30). The prevalence of smoking was similar in both groups. The mean score of the SNOT-22 questionnaire in the study group was 33.06 ± 14.97 (see Table 1). When looking at sinonasal symptoms, 27 out of the 47 patients had nasal obstruction, 23 had nasal discharge, 17 had post-nasal drip and 13 presented with facial pain or headache. Regarding the nasal findings on endoscopy, 24 out of the 47 patients had septal deviation, 30 had inferior turbinate hypertrophy and 6 had nasal polyps.

**Table 1.** Demographic characteristics of the study population

Demographic data	Cases (*n* = 47)	Controls (*n* = 30)	*p* value
Gender (male:female ratio)	3.27	2.75	0.833
Age (mean ± SD; years)	40.3 ± 13.8	47.2 ± 17.2	0.059
Smoking (*n* (%))	22 (46.8)	14 (46.7)	0.990
Sinonasal symptoms (*n* (%))			
– Nasal obstruction	27 (57.4)	0	<0.001
– Nasal discharge	23 (48.9)	0	<0.001
– Post-nasal drip	17 (36.2)	0	<0.001
– Facial pain and/or headache	13 (27.6)	0	<0.001
Endoscopic findings (*n* (%))			
– Nasal polyps	6 (12.8)	0	<0.001
– Septal deviation	24 (51.1)	0	<0.001
– Inferior turbinate hypertrophy	30 (63.8)	0	<0.001

SD = standard deviation

#### The prevalence of laryngeal muscle tension in patients with sinonasal diseases

Among patients with sinonasal diseases, 29 patients (61.7 per cent) had at least one muscle tension pattern compared with only 9 (30 per cent) among the controls (*p* = 0.007). The most common muscle tension pattern observed in the study and control groups was muscle tension pattern II (42.5 *vs* 23.3 per cent, respectively) followed by muscle tension pattern III (38.3 *vs* 20 per cent, respectively). Almost 1 out of 5 patients in the study group had both muscle tension pattern II and muscle tension pattern III in comparison with 13.3 per cent of patients in the control group. It is worth noting that none of the patients and controls had evidence of muscle tension patterns I or IV (see Table 2).

**Table 2.** Prevalence of laryngeal muscle tension pattern in study group and controls

Muscle tension pattern	Patients (*n* = 47)	Controls (*n* = 30)	*p* value
I (*n* (%))	29 (61.7)	9 (30)	0.007
II (*n* (%))	20 (42.5)	7 (23.3)	
III (*n* (%))	18 (38.3)	6 (20)	
II and/or III (*n* (%))	9 (19.1)	4 (13.3)	

When categorised by the nature of the sinonasal disease, patients with a structural problem such as a deviated nasal septum were more likely to have an associated laryngeal muscle tension pattern than those without septal deviation (79.2 *vs* 43.5 per cent, respectively, *p* = 0.012). That significant difference in the prevalence of laryngeal muscle tension was not seen in patients with sinonasal diseases with inflammatory disorders such as inferior turbinate hypertrophy and/or nasal polyps in comparison with those without inferior turbinate hypertrophy and/or nasal polyps (60.6 *vs* 64.3 per cent, respectively, *p* = 0.812).

Inter-rater reliability analysis revealed an intra-class correlation coefficient of 0.896, indicating excellent reliability between the two otolaryngologists who evaluated the endoscopic laryngeal findings.

### The prevalence of laryngeal symptoms in patients with sinonasal diseases and in controls

Twenty-five of the study group patients (53.2 per cent) had hoarseness on presentation in comparison with only 3 patients (10 per cent) in the control group (*p* < 0.001). In addition, 25 patients in the study group had throat clearing and 22 had globus sensation and cough in comparison with none in the control group. With respect to laryngeal findings, seven patients in the study group had arytenoid oedema, which was not observed in the control group. No structural vocal fold abnormalities were noted in either the study or control groups.

## Discussion

Laryngeal behaviour during phonation in patients with sinonasal diseases has not been previously reported. The results of this investigation indicate that patients with a history of sinonasal disease had a higher prevalence of laryngeal muscle tension in comparison with healthy subjects with no history of sinonasal disease. Close to two-thirds of the study group had at least one muscle tension pattern in comparison with only one-third in the control group.

The most common laryngeal muscle tension patterns observed during phonation were medialisation of the false vocal fold followed by antero-posterior shortening of the distance between the petiole and inter-arytenoid space. Almost one out of five patients in the study group had two muscle tension patterns. The laryngeal tension observed in our study group is consistent with numerous studies documenting changes in formants frequencies in patients with sinonasal diseases undergoing sinus surgery.

Chen and Metson, in their investigation on the effect of sinus surgery on speech in five patients with chronic sinusitis, reported an increase in first formant amplitude and a decrease in nasal peak amplitude of the spectra in ‘m’ and ‘n’ post-operatively. These changes correlated with a decrease in nasality for the high vowel ‘i’.^30^ In a study on sound spectrography in patients with nasal polyposis, Hong *et al*. reported a decrease in the frequencies of the nasal formants after endoscopic sinus surgery. The authors noted that sound spectrographic analysis is an objective diagnostic tool to assess nasality in subjects with obstruction of the nasal cavity.^10^ Similarly, Hoseman *et al*. reported a decrease in the energy peaks and bandwidths of formants after endoscopic surgery that was commensurate with a perceptual change in the voice quality of 6 out of 21 patients.^19^

The results of our investigation also showed a higher prevalence of hoarseness in the study group in comparison with the control group (53.2 *vs* 10 per cent). More than two-thirds of patients in the study group with at least one muscle tension pattern (79.3 per cent) reported hoarseness in comparison with only 33.3 per cent in the control group. Among patients with both muscle tension patterns II and III, two-thirds of the study group had hoarseness compared with only half of the control group. These findings corroborate previous studies showing increased prevalence of voice disorders in patients with sinonasal diseases.

In a cohort of 63 patients with nasal polyposis and nasal obstruction, Arslan *et al*. reported a Voice Handicap Index-10 (VHI-10) score of 10.47.^25^ Hall *et al*., in their analysis of 510 patients with nasal polyposis, reported a mean VHI-10 score of 17.1 and a SNOT-22 score of 57.6. The authors noted a decrease in these scores following endoscopic sinus surgery.^31^ Milqvist *et al*. investigated the voice changes in 31 patients with allergic rhinitis and found a significantly higher mean VHI score in comparison with the control group in both the pollen season (18.3 *vs* 2.8, respectively) and the non-pollen season (13.9 *vs* 6.0, respectively).^32^ Similarly, Wu *et al*. demonstrated an association between severity of chronic rhinosinusitis and degree of vocal dysfunction and impairment in the voice-related quality of life score.^33^

The high prevalence of laryngeal muscle tension and hoarseness in patients with sinonasal diseases can be attributed to numerous factors, the most important of which are trafficking of secretions from the sinonasal cavities to the larynx and laryngeal desiccation secondary to nasal obstruction. Post-nasal drip, which was noted in 37.77 per cent of our study group, can instigate throat clearing and cough, leading to an increase in laryngeal sensitivity and tension.^34^ Mouth breathing can also result in dryness of the vocal folds, leading to an increase in phonatory threshold pressure, that is, the pressure needed to set the vocal folds into vibration. As a result, patients have to put more effort into talking, which may adversely impact the laryngeal behaviour during phonation.^35^ Indeed the results of this study showed that patients with septal deviation were more likely to develop laryngeal muscle tension than patients without septal deviation.

Another possible cause for the significantly higher prevalence of muscle tension pattern in patients with sinonasal disease and septal deviation is the known association between nasal obstruction and increased muscle activity of accessory inspiratory muscles. Hiyama *et al*. demonstrated heightened electromyographic activity in the suprahyoid and masseteric muscles among individuals experiencing nasal obstruction compared with oral breathers. Their study involved the examination of 10 healthy Japanese males over a 3-hour sleep period.^36^ Similarly, Trevisan *et al*. investigated the accessory inspiratory muscle activity in 38 nasal-breathing adults using surface electromyography, noting increased recruitment during rapid inspiration in comparison with mouth-breathing subjects. This increase in muscle activity was attributed by the authors to aberrant posture and muscular imbalance among nasal breathers.^37^

The use of intranasal corticosteroids as a medical treatment for inferior turbinate hypertrophy can also be implicated in the pathophysiology of the abnormal laryngeal behaviour associated with sinonasal diseases. Although the impact of intranasal steroids on voice has rarely been studied, one can argue that its effect is similar to that of the inhaled form. Studies have shown that corticosteroid inhalers may induce laryngeal inflammation as a result of chemical irritation or by promoting opportunistic infections such as candidiasis. The irritation of the laryngeal surface mucosa causes hoarseness followed by a compensatory laryngeal muscle tension.^38,39^ Other contributing factors include lower airway disease and laryngopharyngeal reflux disease.^40–45^ The aetiological role of each factor could not be analysed given the retrospective nature of our investigation. Future studies controlling for these confounding factors are warranted.

- Patients with sinonasal diseases have hyponasality in comparison with patients with no sinonasal diseases
- Laryngeal muscle tension is highly prevalent in patients with sinonasal diseases
- Patients with septal deviation are more likely to develop laryngeal muscle tension
- Comprehensive voice evaluation can help us better understand the laryngeal behaviour in patients with sinonasal diseases

This study is an addition to the literature showing an association between sinonasal diseases and laryngeal muscle tension and not a causation. However, it has its limitations. One limitation is the small sample size and a second is its retrospective nature, which explains the lack of objective voice outcome measures, such as acoustic and aerodynamic measures. Moreover, potential confounding variables, such as reflux and allergy, which could influence laryngeal muscle tension, were not addressed. Future prospective studies are needed to explore the link between sinus diseases and voice.

## Conclusion

This study shows a higher prevalence of laryngeal muscle tension and hoarseness in patients with sinonasal diseases in comparison with healthy subjects with no history of sinonasal diseases. Comprehensive voice evaluation of patients with sinonasal diseases can help us to better understand the laryngeal behaviour in affected subjects and the pathogenic role of sinonasal diseases in muscle tension dysphonia.
